# Digital smartphone versus analogue methods of measuring orbital implant motility after enucleation

**DOI:** 10.1038/s41433-025-03936-6

**Published:** 2025-07-26

**Authors:** Bhavesh P. Gopal, Stephen Bell, Noha M. Soliman, David I. T. Sia, Andrew W. Stacey, David Carpenter, Mandeep S. Sagoo

**Affiliations:** 1 Ocular Prosthetics Department, Moorfields Eye Hospital, London, UK; 2 Ocupeye Ltd, Warwick, UK; 3 UCL Institute of Ophthalmology, London, UK; 4 NIHR Biomedical Research Centre for Ophthalmology at Moorfields Eye Hospital and UCL Institute of Ophthalmology, London, UK; 5 Department of Ophthalmology, University of Adelaide, Royal Adelaide Hospital, Adelaide, SA Australia; 6 Department of Ophthalmology, Flinders University, Flinders Medical Centre, Bedford Park, SA Australia; 7 Ocular Oncology Service, Moorfields Eye Hospital, City Road, London, UK; 8 Retinoblastoma Service, Royal London Hospital, Whitechapel, London, UK; 9 Department of Ophthalmology, University of Washington, Seattle, WA USA

**Keywords:** Outcomes research, Medical imaging

## Abstract

**Purpose:**

To compare standard rule measurement (gold standard, analogue) method of orbital implant motility against digital image analysis on smart phone measurement software, Medigrid and ImageJ.

**Design:**

Single centre observational, non-randomised, non-masked cohort study in enucleated patients.

**Methods:**

A standard rule measurement (gold standard) was used to measure vertical and horizontal movements of the fellow eye, prosthesis, and orbital implant. At the same time, photographs taken in the same positions of gaze were analysed using ImageJ software (NIH, Bethesda, Maryland, USA) and the smartphone app mediGrid (IRISS Medical Technologies, UK). Bland-Altman plots were used to compare the digital modalities, with the current gold standard.

**Results:**

A total of 54 patients were tested for fellow eye motility, ocular prosthesis and orbital implant motility following primary enucleation. The median time since enucleation was 282 days (range: 45–11,278). Image-J had consistently lower limits of bias and agreement than mediGrid, suggesting that image-J is more equivalent to the standard rule measurement. The largest difference in the limit of agreement between mediGrid and ImageJ can be found most in upgaze and downgaze.

**Conclusions:**

In our analyses, Image-J was found to be a more accurate measure of orbital implant motility, than mediGrid.

## Introduction

Enucleation, the surgical removal of the entire globe from the orbital socket, including transection of the optic nerve [1] is indicated for cases of intraocular tumours, trauma, blind painful eyes, phthisis, congenital anophthalmia, severe microphthalmia, and resistant endophthalmitis [1, 2]. To replace some of the volume lost following enucleation, orbital implants are inserted in the enucleated socket [3, 4]. The rectus muscles can be attached to the conjunctival fornices in the myoconjunctival technique [5] or directly sutured to the implant that has an absorbable covering [6] or a traction suture [7]. The goal is to provide maximum motility and to achieve the best symmetrical cosmesis with the fellow eye [8, 9]. It will also provide a tissue bed to impart direct motion to the ocular prosthesis [10]. Implant choice varies and can be categorised into non-integrated (non-porous) such as polymethyl methacrylate (PMMA) orbital implant and integrated (porous) such as hydroxyapatite (HA) orbital implant [5].

There are several described methods of motility measurement and ocular prosthesis motility can be described only using subjective evaluation by the ocularist or patient. Several techniques for objective assessment of ocular prosthesis motility have been described which include: amplitude measurement with an arc perimeter [11], Kestenbaum limbus test [12], magnetic search coil [13], prism and digital photograph [8], infrared oculography [14] and slit lamp and rule [15]. The reliability and reproducibility of these tests has not been reported. Recently, a gaze and pupil-tracking system “iView X” was used for measuring horizontal motility of the ocular prosthesis and the contralateral eye, with high reliability and reproducibility [16]. This device consisted of 2 computers and 3 monitors connected to each other through a direct network connection; however, it had an inadequate measurement in 36% of patients [16]. Sparse attention has been given to just evaluating orbital implant motility [5, 17].

Digital technology has now advanced with smart phone software capable of rapid accurate measurements, even allowing home measurement of ocular alignment [18]. In the current report, we evaluated orbital implant motility following enucleation with the gold standard clinical technique of standard rule measurement (SRM) against digital measurement using two smartphone photographic programmes.

## Methods

This was a single centre observational, non-randomised and non-masked cohort study in enucleated patients attending their routine visits to the ocular prosthetics department at Moorfields Eye Hospital. Ethical approval was obtained from Research Ethics Committee (reference 18/LO/1990) and the study adhered to the tenets of the Declaration of Helsinki.

### Patient recruitment

All patients who attended the ocular prosthetics department at Moorfields Eye Hospital for routine follow up were offered recruitment by the chief ocularist or his delegated staff. Inclusion criteria included adult patients who had undergone enucleation, performed with an orbital implant technique with recti muscles sutured directly to a mesh around the implant in an anterior location. Exclusion criteria included a history of other socket procedures such as radiotherapy to socket, implant exposure repair, secondary orbital implant, concurrent socket pathology such as socket infection or exposure, giant papillary conjunctivitis, systemic chemotherapy, presence of ocular motility disorders on either side of the face, muscle restrictive disorders, previous trauma, idiopathic orbital inflammatory disease and neurological disorders such as cranial neuropathies or multiple sclerosis.

### Measurement of ocular motility

**Fig. 1:**
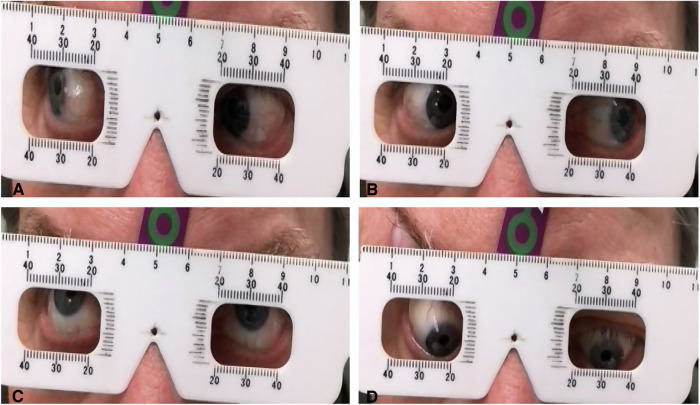
Photographs to show measurement technique for the gold standard standard rule measurement (SRM). Orbital implant movements were measured using standard ruler in fixed position, showing **A** movement in right-gaze, **B** movement in left-gaze, **C** movement in up-gaze, and **D** movement  in down-gaze.

**Fig. 2:**
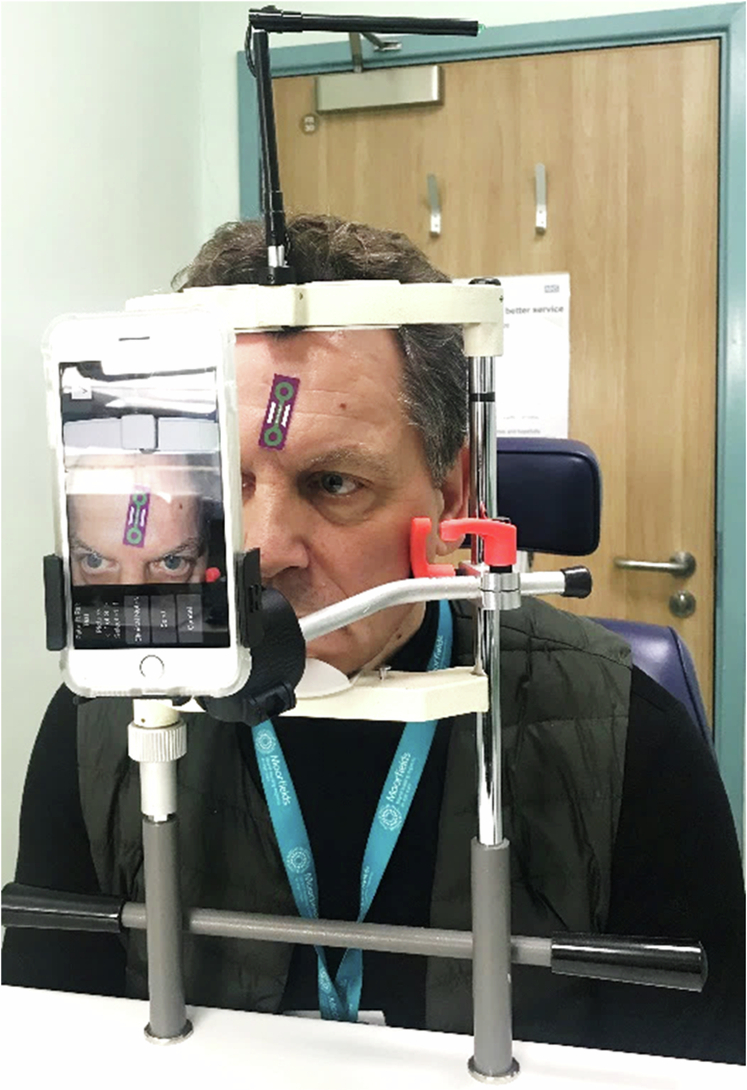
Photograph of modified slitlamp apparatus to standardise the position of the subject and the smartphone for ocular movement studies. A medically compliant sticker placed on patient’s forehead provides the calibration for the smartphone app mediGrid. Colour photographs taken were analysed for movements with mediGrid as well as with ImageJ software.

Measurements of movement were made for the fellow eye, the prosthesis and the orbital implant. The motility of each of these was tested in three ways. A standard ruler measurement (SRM) of excursion was considered the gold standard (Fig. 1) and compared against two photographic measurement methods using Image J software (NIH, Bethesda, Maryland, USA) and the smartphone app (Fig. 2), mediGrid (IRISS Medical Technologies, UK).

A small dot mark was placed in the midline of the bridge of the nose (mid canthal distance) of the patient using a pen-marker. This dot was used as a reference point for measurements. The measurement point for the normal eye was the centre of the pupil, for the prosthesis was the centre of the painted pupil and for the implant was a dot marked by surgical marker pen in the centre of the socket over the implant.

The patient was instructed to sit upright, keep the head still in the primary position in a modified slitlamp apparatus, and follow a series of instructed gaze directions, with a 1 mm scale ruler (SRM) held in the same plane of the eye. The degree of movement was calculated by measuring the distance from the midline dot reference to the measurement point in straight-gaze and deducting this from the measurement of the midline dot to the measurement point in another gaze position. The instructed gaze directions were up, down, right and left-gaze. In order to compare right and left eyes simultaneously, these measurements were translated to up, down, adduction, and abduction. On down-gaze, the upper eyelids were raised by an assistant. A photograph was taken of each excursion (Fig. 1).

For the smartphone methods, at the same time, the patient had a sticker with a scale placed on their forehead or cheek in a plane as close as possible to the eye. This sticker served as a scale for the mediGrid app to overlay a calibrated grid on a photograph. At each gaze direction, images were captured using a smartphone camera to capture images of the patient’s eye movements. This image was then analysed for excursion by ImageJ software and mediGrid. The mediGrid app transferred the selected images to a centralised processing server where a scale calibrated grid was overlaid onto the photograph, allowing the excursion to be read from the scale. Measurement of the distance using the software ImageJ was done in 2 ways: using the markings on the ruler to calibrate the number of pixels in the photograph and using this calibration to calculate the distance; the other was using the overlaid grid from the mediGrid app to calibrate the number of pixels in the photograph, and using this calibration to calculate distance (Fig. 2).

Bland-Altman plots were created to compare the Medigrid and Image J measurement platforms versus the gold standard (SRM). A separate plot was created for each imaging modality (Medigrid vs. SRM, ImageJ vs. SRM) as well as each direction (Up, Down, ADDuction, ABduction). Bland-Altman plots analysed the bias between mean difference in the imaging modality and the standard rule measurements to estimate an agreement interval. The limits of agreement (LOA) are equivalent to confidence intervals, within 95% of data differences lies within one limit of agreement above or below the data midpoint. If the bias is negative, then the imaging modality on average has a higher value than the gold standard value. The x-axis will show the mean of ImageJ/Medigrid and the gold standard. The y-axis will show the difference between ImageJ/Medigrid and the gold standard.

## Results

### Patient demographics

Out of 54 patients (*n* = 54) who qualified for this study, 29 were female (53.7%) and 25 were male (46.2%). The median time since enucleation was 282 days (range: 45–11,278). There were 24 right eyes (44.44%) and 30 left eyes (55.55%).

**Fig. 3:**
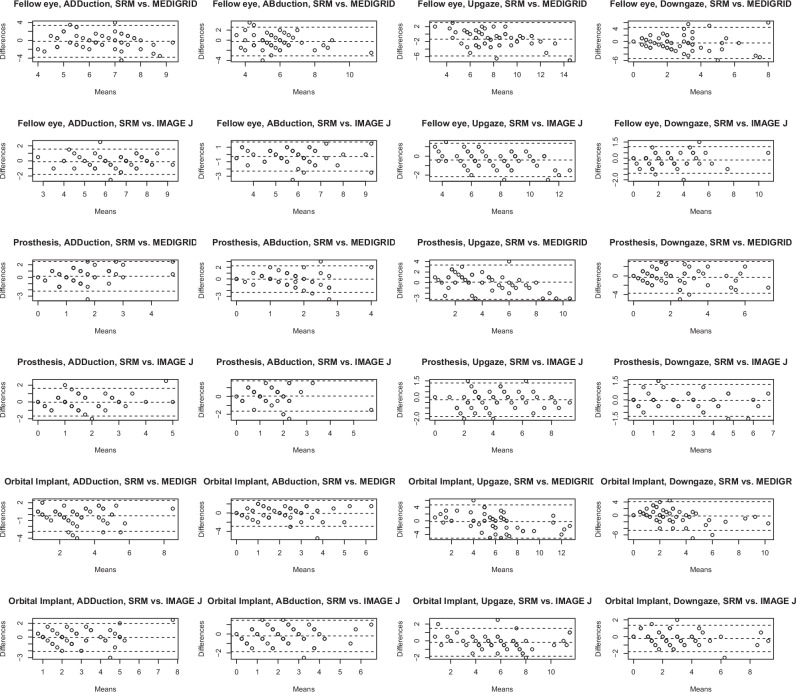
Bland Altman plots of the 4 postions studies of ADDuction, ABduction, upgaze and downgaze, in fellow eye, prosthesis and orbital implant of standard ruler measurement (SRM) versus mediGrid or ImageJ. If the bias is negative, then the imaging modality on average has a higher value than the gold standard value. The x-axis shows the mean of ImageJ/Medigrid and the gold standard. The y-axis shows the difference between ImageJ/Medigrid and the gold standard.

Two digital methods were compared to the gold standard, a standard rule measurement. Analysis was carried out using Bland-Altman plots in each gaze direction (adduction, abduction, upgaze, downgaze). A separate plot was created for each imaging modality (Medigrid vs. SRM, ImageJ vs. SRM) as well as each direction (Up, Down, ADDuction, ABduction) (Fig. 3).

### Movement of the fellow eye

The fellow eye had a mean SRM of 6.3 mm in adduction, 5.6 mm in abduction, 7.4 mm in upgaze and 2.6 mm in downgaze. Comparison of MediGrid versus SRM and ImageJ versus SRM showed a negative bias, meaning that these modalities overestimated the measurement. ImageJ was closer to the SRM measurement (Table 1).

**Table 1:** Comparison of measurements of fellow eye, prosthesis and orbital implant motility taken by standard ruler measurement (SRM) versus mediGrid or ImageJ in the 4 positions of gaze.

			ADDuction	Abduction	Upgaze	Downgaze
Fellow eye		Mean SRM measurment	6.3	5.6	7.4	2.6
	MediGrid vs. SRM	Bias	−0.2	−0.3	−1.4	−0.5
		Limit of agreement	3.6	2.8	4.5	4.9
	Image J vs. SRM	Bias	−0.1	−0.3	−0.4	−0.2
		Limit of agreement	1.7	2.0	1.8	1.2
Standard prosthesis		Mean SRM measurment	1.6	1.5	4.0	2.1
	MediGrid vs. SRM	Bias	0.2	−0.04	0.1	−0.3
		Limit of agreement	2.4	2.3	3.2	3.4
	Image J vs. SRM	Bias	−0.04	0.05	−0.2	−0.05
		Limit of agreement	1.6	1.7	1.6	1.3
Orbital implant		Mean SRM measurment	2.8	2.1	5.7	2.7
	MediGrid vs. SRM	Bias	−0.2	−0.1	−0.2	−0.3
		Limit of agreement	2.6	2.8	4.9	4.4
	Image J vs. SRM	Bias	−0.1	−0.2	−0.2	−0.2
		Limit of agreement	2	1.7	1.7	1.6

“Bias” represents the mean of the differences between the two measurement modalities in all patients.

“Limit of agreement” is a measure of confidence: 95% of data fall within Bias +/− Limit of Agreement.

### Movement of the prosthesis

Motility of the prosthesis had a mean SRM of 1.6 mm in adduction, 1.5 mm in abduction, 4.0 mm in upgaze and 2.1 mm in downgaze. Comparison of MediGrid versus SRM had a negative bias (overestimation) for abduction and downgaze, but a positive bias (underestimation) for adduction and upgaze. ImageJ versus SRM showed a negative bias, for all positions except abduction (Table 1).

### Movement of the orbital implant

Motility of the orbital implant had a mean SRM of 2.8 mm in adduction, 2.1 mm in abduction, 5.7 mm in upgaze and 2.7 mm in downgaze. Comparison of MediGrid versus SRM and ImageJ versus SRM showed a negative bias (Table 1).

### Comparison of analogue versus digital measurements

Both ImageJ and Medigrid demonstrated small bias, indicating accuracy of both tests, but mostly a small overestimation. However, Image J demonstrated consistently lower limits of agreement, which signifies that it is more precisely estimating the SRM. The difference in the limits of agreement is consistent with the ImageJ limits of agreement often half as large as the Medigrid limits of agreement (Table 1).

## Discussion

Ocular prosthesis motility is a crucial outcome for enucleation surgery in the treatment of an anophthalmic patient as it remains the single most objective measure of success that provides confidence in professional and personal interactions as well and has social acceptability [19].

The aim of this study was to compare the two digital measurement tools, Image-J and Medigrid with the standard rule measurements. A significant finding was that Image-J was found to measure orbital implant motility more accurately. In all directions of gaze, Image-J had narrower limits of agreement to Medigrid. Image-J had a lower bias compared to Medigrid for adduction and downgaze. Image-J had a greater bias compared to Medigrid for abduction (−0.2 vs −0.1). Whilst both imaging modalities had an identical bias for upgaze at −0.2.

The big question arising from this study is whether Image-J can replace the standard rule measurements. This is desirable for digital record keeping as well as for studying the movements of different implant and prosthesis types. Whilst Image-J is a quick method of measuring orbital implant motility and is able to digitise data storage, it is still an inferior method to the standard rule but was superior to the other platform tested. Medigrid calculated orbital implant motility by overlaying a grid over the image of the patient’s face. Further work is needed to improve software calibration that produces the grid, to increase accuracy. It is possible that Medigrid is less accurate as the calibration sticker is difficult to place in the same plane as the eye.

Only one study has directly compared measuring modalities. Kupersmith et al. [20] compared the Kestenbaum limbus test, the adapted cervical range of motion and a grading scale of 0 to −5 in 40 patients using two examiners. The cervical range of motion measures degrees of ocular movement and the grading scale measures 0 for full excursion and −5 for failing to reach the midline. The cervical range of motion and Kestenbaum limbus test had similar inter-examiner repeatability whilst the grading scale had an identical inter-examiner repeatability.

### Limitations

The sample size was small, which may have affected the reliability of the measurements. Also, one investigator was assigned for data collection and processing to add reliability to results. Unintentional selection and observer bias cannot be eliminated. Moreover, between measurements the patient may move their head from the headrest, altering the angle of their head. This may have altered the measured orbital implant motility. Additionally, we have made an assumption that the standard rule measurement is the gold standard that uniformly measures eye movement for each patient. However, differences in the angle of how the rule is held can alter the calculated amount of motility. Furthermore, whilst measuring downgaze, the investigator raises the patient’s upper eyelids to stop obscuration of the upper eyelids at front gaze. However, this may not be done in an identical way for each patient, which could add variation to data.

## Conclusion

In conclusion, in this study, we demonstrate that digital apps on a smartphone are capable of measuring implant and prosthesis motility. From the digital smartphone measurement tools tested here, Image-J is superior to Medigrid in measuring orbital implant motility. However, the standard rule measurements method is still the gold standard. As a method of digitising data of orbital implant motility, Image-J is a reliable technique. This will spur further investigation of motility of different types of implant and prosthesis. More research is also needed to improve the software calibration that produces the grid of the Medigrid technique, to further increase accuracy.

## Summary

### What was known before:

- Enucleation often necessitates the insertion of orbital implants to replace lost volume and improve ocular prosthesis motility, critical for patient outcomes.
- Various methods for assessing ocular prosthesis motility exist, but their reliability and reproducibility have not been well-documented.
- Traditional methods like standard rule measurement (SRM) are considered the gold standard, while newer digital methods using smartphone software have been less explored in clinical settings.

### What this study adds:

- This study compares SRM with digital image analysis using ImageJ and mediGrid software, identifying ImageJ as a more accurate alternative to SRM for measuring orbital implant motility.
- Digital measurement methods, particularly with ImageJ, show promise for providing reliable, accessible, and potentially home-based assessment tools for postoperative care in enucleated patients.
- The findings suggest that smartphone-based technologies could supplement or replace traditional analogue methods, improving the ease and accuracy of motility assessments.

## Supplementary information

Eye Reporting Checklist

## Data Availability

All relevant raw data and materials described in this manuscript will be made freely available to researchers upon reasonable request for non-commercial purposes, provided that participant confidentiality is maintained. Data supporting the findings of this study can be accessed upon request from the corresponding author.
